# Diagnostic Accuracy of Point-of-Care HCV Viral Load Assays for HCV Diagnosis: A Systematic Review and Meta-Analysis

**DOI:** 10.3390/diagnostics12051255

**Published:** 2022-05-18

**Authors:** Weiming Tang, Yusha Tao, Emmanuel Fajardo, Elena Ivanova Reipold, Roger Chou, Joseph D. Tucker, Philippa Easterbrook

**Affiliations:** 1 University of North Carolina Project-China, Guangzhou 510095, China; yusha.tao@seshglobal.org (Y.T.); jdtucker@med.unc.edu (J.D.T.); 2 Institute for Global Health and Infectious Diseases, University of North Carolina at Chaple Hill, Chapel Hill, NC 27599, USA; 3 Department of Global HIV, Hepatitis and STI Programmes, World Health Organization, 1211 Geneva, Switzerland; fajardoe@who.int (E.F.); philippa.easterbrook@hotmail.com (P.E.); 4 FIND, The Global Alliance for Diagnostics, 1202 Geneva, Switzerland; elena.ivanova@finddx.org; 5 Departments of Medicine, Oregon Health & Science University, Portland, OR 97239, USA; chour@ohsu.edu

**Keywords:** diagnostic accuracy, hepatitis c, point-of-care, viral load testing

## Abstract

Despite the widespread availability of curative treatment with direct-acting antivirals, a significant proportion of people with HCV remain undiagnosed and untreated. New point-of-care (PoC) HCV RNA assays that can be used in clinical settings may help expand access to testing and treatment. This study aimed to evaluate the diagnostic performance of PoC HCV viral load assays compared to laboratory-based testing. **Methods:** We searched three databases for studies published before May 2021 that evaluated PoC HCV RNA assays against a laboratory NAT reference standard (Prospero CRD42021269022). Random effects bivariate models were used to summarize the estimates. Stratified analyses were performed based on geographic region, population (PWID, etc.), and specimen type (serum/plasma or fingerstick; fresh or frozen). We used the GRADE approach to assess the certainty of the evidence. **Results:** A total of 25 studies were eligible. We evaluated five different commercially available viral load assays. The pooled sensitivity and specificity were 99% (95% CI: 98–99%) and 99% (95% CI: 99–100%), respectively. High sensitivity and specificity were observed across different assays, study settings (including LMICs and HICs), and populations. There was a small but statistically significant reduction in sensitivity for fingersticks compared to serum or plasma samples (98% vs. 100%, *p* < 0.05), but the specificity was similar between frozen and fresh samples. The evidence was rated as moderate-high certainty. **Conclusions:** PoC HCV viral load assays demonstrate excellent diagnostic performance in various settings and populations. The WHO now recommends using PoC HCV viral load assays as an additional strategy to promote access to confirmatory viral load testing and treatment.

## 1. Introduction

Chronic HCV infection is a significant global health issue. An estimated 58 million individuals live with chronic hepatitis C virus (HCV) infection, and there were around 300,000 deaths from HCV-related cirrhosis or hepatocellular carcinoma in 2019 [1]. The World Health Organization (WHO) has set ambitious goals for eradicating viral hepatitis as a public health issue by 2030, including a 90% reduction in new HCV infections and a 65% reduction in HCV-related deaths [2,3]. Achieving these impact targets requires 90% of people living with HCV to be diagnosed and 80% of those eligible to be treated. However, as of 2019, only 20% of people living with HCV worldwide were aware of their HCV status, and 13% had been treated [4]. Diagnosis rates are even lower in low-income countries (LMICs), with only 8% of people living with HCV diagnosed in 2016, compared to 43% in high-income countries [4]. Addressing this significant testing and treatment gap to achieve the WHO targets requires a substantial scale-up of testing and treatment with simplified decentralized service delivery models and task-shifting [5].

Chronic HCV infection is diagnosed in two steps, with an initial screening step using HCV antibody serological tests to ascertain prior exposure to HCV and a confirmatory step using laboratory-based HCV nucleic acid RNA tests or core antigen (HCVcAg) tests to determine the presence of active viremic HCV infection and the need for treatment [6]. However, access to these laboratory-based assays is limited in many resource-limited settings. The lack of viral load confirmation means that many of those with chronic HCV infections are never linked to care and treatment. Overall, HCV RNA point-of-care viral load assays are easier to use than the laboratory-based NAT assays and may improve access to the diagnosis of viremic HCV infections, followed by treatment and monitoring of the treatment response [7]. In general, these point-of-care (PoC) devices are battery-operated and are not dependent on continuous electricity to run; in addition, they use reagents that do not require refrigeration and be stored at ambient temperatures. In this study, we defined PoC testing as testing performed near or at the site of patient care [8]. Testing procedures use a fingerstick sample or whole blood, and the result is available within 2 h to inform clinical decision making. There are five different commercial assays: the Xpert HCV Viral load assay (Cepheid, Sunnyvale, CA, USA), the Xpert HCV VL Fingerstick assay (Cepheid, Sunnyvale, CA, USA), the Genedrive HCV ID Kit (Genedrive, Manchester, UK), the Truenat HCV RNA assay (Molbio Diagnostics, Goa, India), and the SAMBA II HCV Qualitative Whole Blood Test (Diagnostics for the New World, Birmingham, UK).

This study aims to evaluate the diagnostic accuracy of PoC assays compared to the standard of care laboratory-based HCV VL assays. This study complements a related systematic review to assess the impact of using PoC HCV VL on the uptake of HCV RNA testing and HCV treatment and the turnaround time to treatment initiation in HCV antibody-positive people in different population subgroups and settings. The findings from both reviews were used to inform recommendations on approaches to viral load testing in updated WHO guidelines on chronic hepatitis C infection.

## 2. Methods

### 2.1. Research Question

The study protocol was registered on PROSPERO (registration number CRD42021269022) [9]. This study was reported following PRISMA guidance (Supplementary Table S1) [10]. The research question was “In adults identified for HCV testing (P), what is the diagnostic accuracy (O; sensitivity and specificity) of point-of-care HCV RNA assays (I) versus laboratory-based HCV Nucleic acid testing (C)?”, structured in a PICO format.

### 2.2. Search Strategy

We searched three biomedical databases (Medline, Embase, and Google Scholar) on 31 May 2021. To minimize publication bias, an additional search was performed for relevant conference abstracts from 2018 to 2020. Manual searches of the bibliographic references of relevant full-text papers were also performed to supplement the electronic searches. We also included unpublished data from a multicenter evaluation conducted by FIND on the Truenat HCV RNA assay (Molbio Diagnostics, India) in five countries.

The medical subject headings (MeSH)-accessible text terms and subject headings used to conduct the searches included “HCV” or “hepatitis C virus”; “point-of-care” or “PoC” or “rapid diagnostic” or “near-patient”; “RNA” or “viral load” or “PCR” or “NAT.” Further details of the search strategy can be found in Supplementary Table S2.

### 2.3. Selection Criteria

Studies were included if they met the following criteria:

(1) The study evaluated the diagnostic accuracy of any PoC HCV RNA assays against an acceptable quality-assured laboratory reference for HCV RNA determination, including Abbott RealTime HCV (Abbott Molecular, Des Plaines, USA), Generic HCV Charge Virale (Biocentric, Bandol, France), COBAS AmpliPrep/COBAS TaqMan HCV Qualitative or Quantitative Tests, Version 2.0 (Roche Molecular Diagnostics, Santa Clara, CA, USA), APTIMA HCV Quant Dx Assay (Hologic Inc., Marlborough, MA, USA), Artus HCV RG RT-PCR or QS-RGQ (Qiagen, Hilden, Germany), and HCV Real-TM Quant Dx (Sacace Biotechnologies, Como, Italy). These laboratory-based HCV virological tests are all commercially available products and have obtained CE mark certificates.
(2) The study used a commercial PoC HCV viral load nucleic acid assay for point-of-care or near-patient nucleic acid testing. Assays include the Xpert HCV Viral load and the Xpert HCV VL Fingerstick assays (Cepheid, Sunnyvale, CA, USA), the Genedrive HCV ID Kit (Genedrive, Manchester, UK), the Truenat HCV RNA assay (Molbio Diagnostics, Goa, India), and the SAMBA II HCV Qualitative Whole Blood Test (Diagnostics for the New World, Birmingham, UK). These PoC HCV virological tests are all currently available in the market and have obtained CE mark certificates.
(3) The study evaluated adults (>18 years of age), including key populations at high risk of HCV infection.
(4) The study reported diagnostic accuracy or provided data to allow the calculation of diagnostic accuracy (True Positive [TP], False Positive [FP], True Negative [TN], and False Negative [FN]).

There were no restrictions on study design; case-control studies, cross-sectional studies, and cohort studies were all included.

### 2.4. Data Extraction

Two investigators independently reviewed all citations for potential eligibility. Full-text articles were obtained for potentially eligible citations and underwent independent dual review for final inclusion. Disagreements were resolved by consensus or with the input of a third investigator. Extraction of the data was done by one reviewer and checked by a second reviewer. We retrieved data on the author, year, country, recruitment setting (HCV clinics, drug treatment sites, or unreported), nature of the study population (known HCV positive or unknown HCV status (people injecting drugs, other high-risk populations, and the general population)), study design (case-control study, cross-sectional study, or cohort study), sample size, specimen type (serum/plasma or fingerstick, fresh or frozen), and type of PoC assays used. Outcomes were defined as the sensitivity and specificity of the index PoC tests. If eligible studies had missing or incomplete data, corresponding authors were contacted by email.

### 2.5. Outcome and Analysis

Data were extracted to construct diagnostic 2 × 2 contingency tables (PoC index test results versus laboratory-based results) to assess the sensitivity and specificity measures. Random effect bivariate models were used to summarize the sensitivity and specificity and describe the variability in test performance across studies. A forest plot was generated from the accuracy parameters to display the point estimates and 95% CI for each study and the pooled estimates. The I-square for the assessment of statistical heterogeneity was used; however, this measure is difficult to interpret in diagnostic accuracy meta-analyses because neither it nor the other standard methods for measuring statistical heterogeneity take into account threshold effects [11].

### 2.6. Risk of Bias Assessments

Authors YT and MC assessed the risk of bias for each study using methods adapted from the QUADAS-2 tool [12,13]. Where there was a disagreement between the two overall risk of bias assessments by the initial assessors, a third author, WT, was consulted.

### 2.7. Grading Quality of Evidence

The quality of evidence was assessed using the GRADE framework [14,15]. The quality of evidence assessment was based on the following domains: overall risk of bias across studies, consistency of results across studies, the precision of the estimate of effect, and directness of comparisons.

For imprecision, we deducted one level if the difference between the upper and lower limits of the pooled CI was greater than 10%; for reporting bias, we deducted one level if reporting bias was suspected; for risk of bias, we deducted one level for overall moderate risk of bias and two levels for overall high risk of bias; for consistency, we deducted one level if more than 75% of studies did not report point estimates within a 10% range; and for indirectness, we degraded one degree if there was any indirectness in populations or outcomes. The review was restricted to commercially available PoC assays. We did not downgrade for indirectness in outcomes because the purpose of the review was to assess the certainty of accuracy measures [16]. Based on our assessments of these domains, we graded the certainty for each body of evidence as high, moderate, low, or very low. The evidence started as high; we ended with a final assessment based on the described domain assessments.

## 3. Results

### 3.1. Study Characteristics

**Figure 1:**
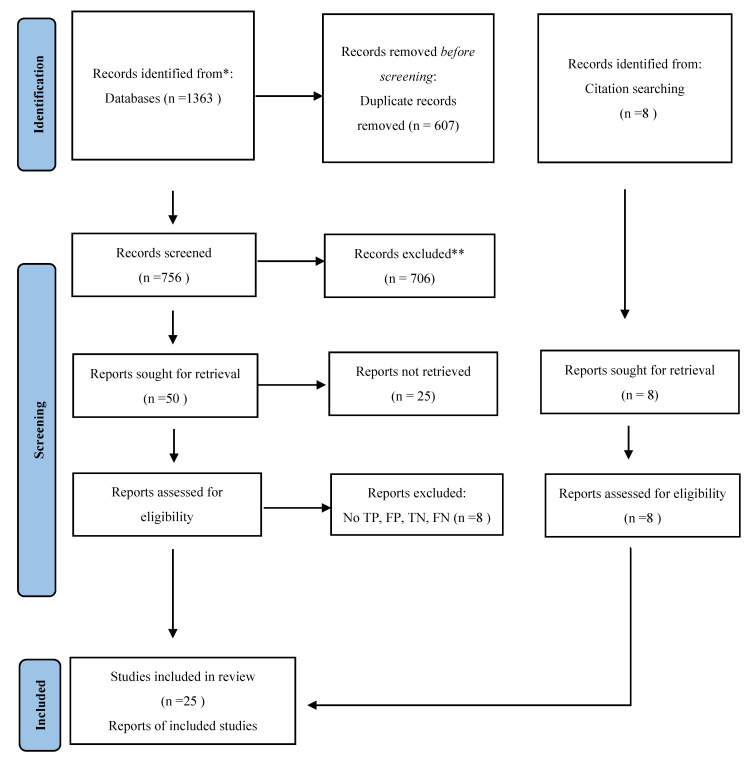
* Consider, if feasible to do so, reporting the number of records identified from each database or register searched (rather than the total number across all databases/registers). ** If automation tools were used, indicate how many records were excluded by a human and how many were excluded by automation tools. From: http://www.prisma-statement.org/ (accessed on 1 September 2021).

**Figure 2:**
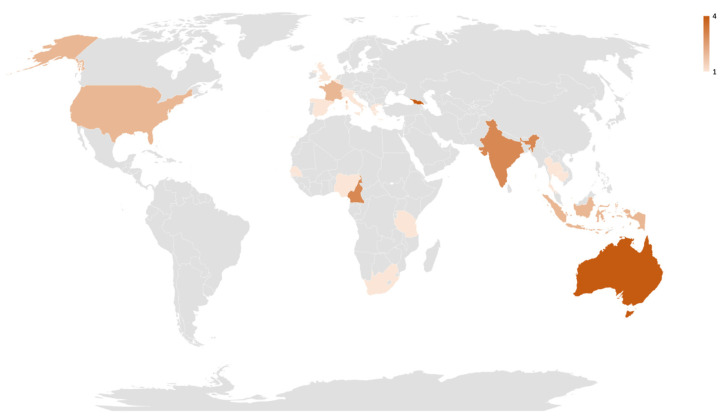
Geographic distribution of countries from 25 studies included within the review (shaded in red). **WHO regions: Africa**—Cameroon (*n* = 3), Nigeria (*n* = 1), Senegal (*n* = 1), South Africa (*n* = 1), and Tanzania (*n* = 1)**. Americas**—the United States (*n* = 2)**. Europe**—Belgium (*n* = 1), France (*n* = 2), Georgia (*n* = 4), Greece (*n* = 1), Italy (*n* = 1), Spain (*n* = 1), Switzerland (*n* = 1), UK (*n* = 1), and Ukraine (*n* = 2)**. South-east Asia**—India (*n* = 3), Indonesia (*n* = 2), and Thailand (*n* = 1)**. West Pacific**—Australia (*n* = 4), Cambodia (*n* = 1), and Malaysia (*n* = 1).

The flow diagram for study inclusion is shown in Figure 1. We identified 1371 citations, and 25 [17,18,19,20,21,22,23,24,25,26,27,28,29,30,31,32,33,34,35,36,37,38,39,40,41] met the criteria for inclusion in the meta-analysis. Of the 25 included studies, 13 were published before 2020. Twelve studies were conducted in high-income countries and thirteen in LMICs (Figure 2). Sixteen studies evaluated the Xpert HCV Viral load assay, six studies the Genedrive assay, two studies the Truenat assay, and one study the SAMBA II assay. Concerning sampling methods, eleven studies used fingerstick capillary whole blood, ten studies used serum or plasma, and four used venous whole blood. Sixteen studies used fresh samples, and nine used frozen samples. Most of the studies included in this review were conducted in PoC settings; for example, twelve studies were conducted at HCV clinics and eight at drug treatment sites for people who inject drugs (PWID). The sample volumes of the evaluated PoC viral load assays ranged from 30 µL to 1000 µL, and the reported manufacturers’ limits of detection/thresholds ranged from 10 IU/mL to 2362 IU/mL.

### 3.2. Quality Assessment

**Figure 3:**
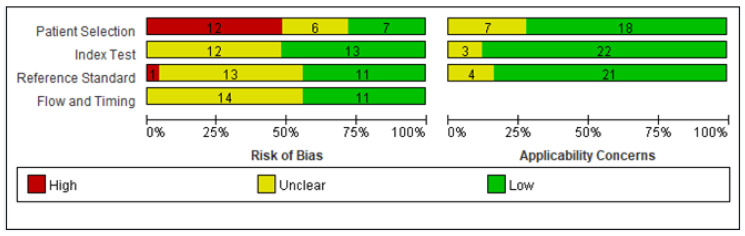
Quality assessment of Diagnostic Accuracy Studies 2 (QUADAS-2) study quality summary.

The QUADAS-2 tool was used for quality assessment. Figure 3 summarizes the risk of bias assessment; a tabular summary is available as a Supplementary Figure S1. All studies scored as having a high risk of bias based on study design (i.e., case-control study), but most were scored as low risk or unclear risk based on other assessment categories.

### 3.3. Overall Diagnostic Accuracy

**Figure 4:**
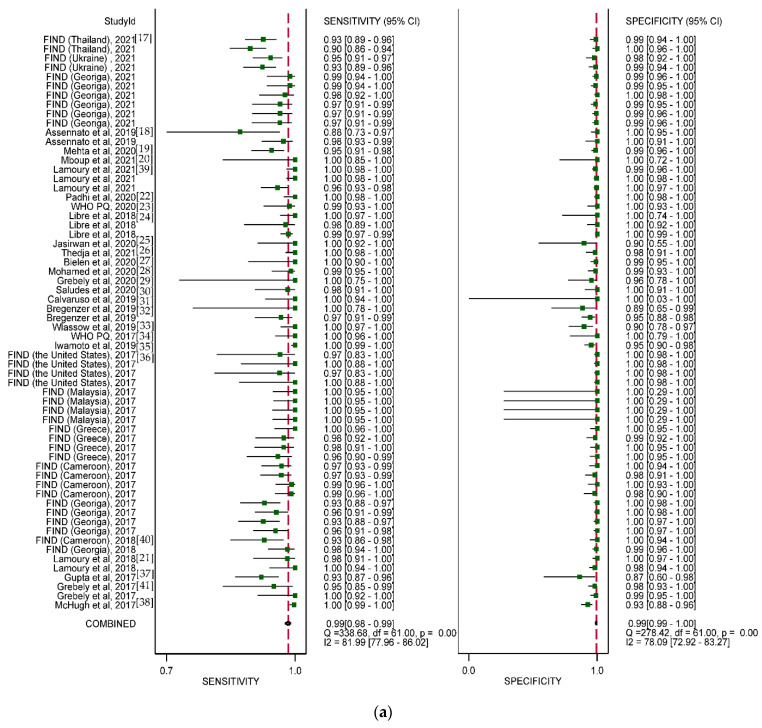

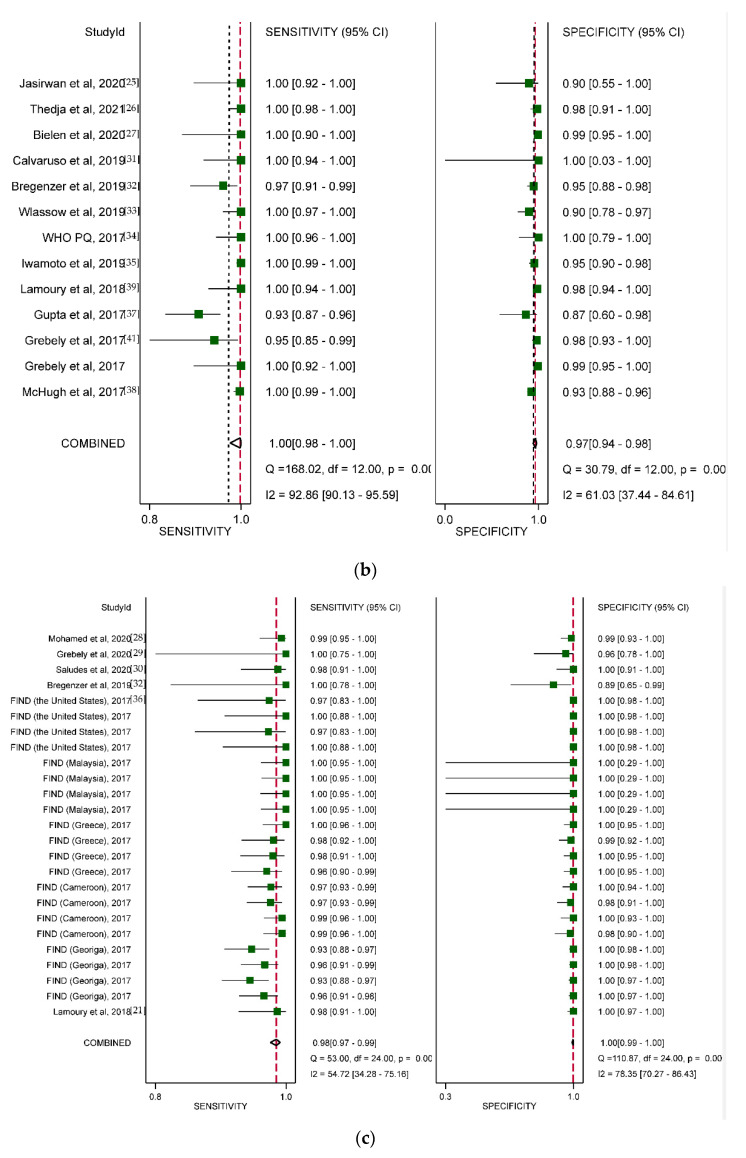

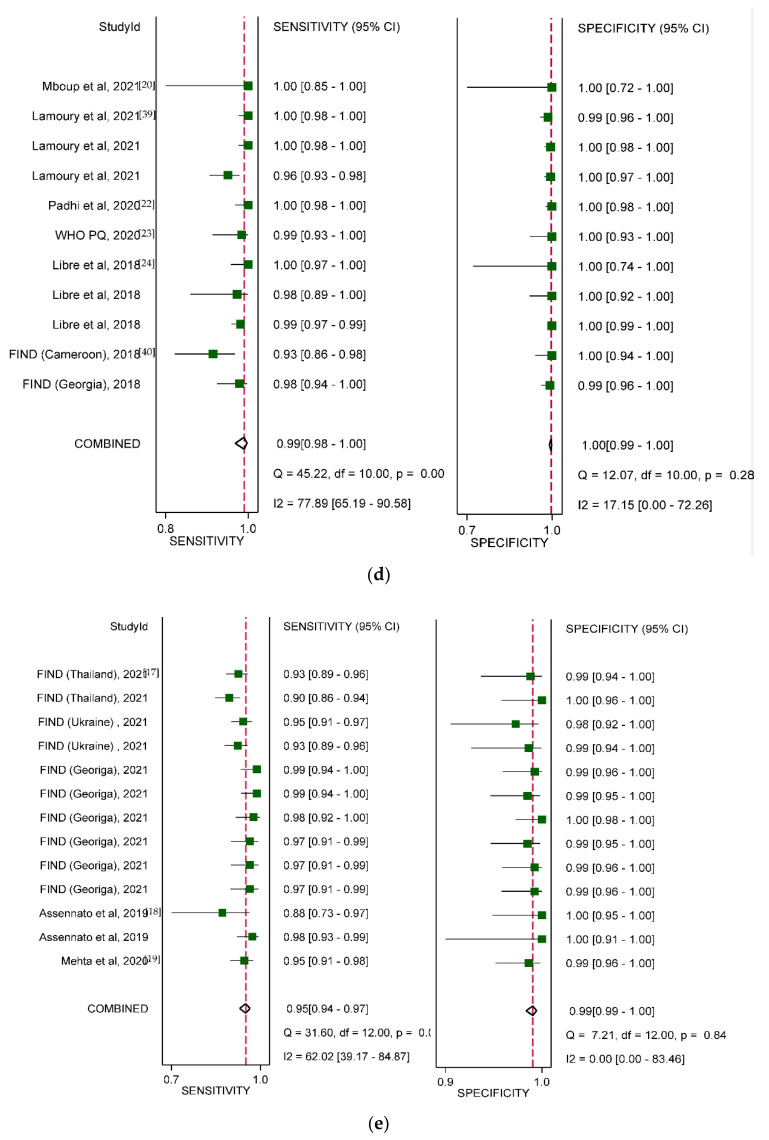
(**a**) Meta–analysis of diagnostic accuracy (sensitivity and specificity) for point–of–care HCV RNA assays for the diagnosis of chronic HCV infection (62 data sets from 25 studies). (**b**) Meta–analysis of diagnostic accuracy (sensitivity and specificity) for point–of–care HCV RNA assays for the diagnosis of chronic HCV infection (Xpert HCV Viral Load) (13 data sets from 12 studies). (**c**) Meta–analysis of diagnostic accuracy (sensitivity and specificity) for point–of–care HCV RNA assays for the diagnosis of chronic HCV infection (Xpert HCV VL Fingerstick) (25 data sets from 6 studies). (**d**) Meta–analysis of diagnostic accuracy (sensitivity and specificity) for point–of–care HCV RNA assays for the diagnosis of chronic HCV infection (Genedrive HCV ID kit) (11 data sets from 6 studies). (**e**) Meta–analysis of diagnostic accuracy (sensitivity and specificity) for point–of–care HCV RNA assays for the diagnosis of chronic HCV infection (Truenat and SAMBA) (13 data sets from 3 studies).

A total of 62 evaluations of 5 different HCV PoC RNA assays from 25 different studies were analyzed. Across all assays, the pooled sensitivity and specificity were 99% (95% CI: 98–99%) and 99% (95% CI: 99–100%), respectively (Figure 4a).

### 3.4. Diagnostic Accuracy by Type of HCV VL PoC Assay

**Xpert HCV viral load:** Sixteen studies (20 data sets) based on 3941 samples collected between 2012 and 2019 were included in the meta-analysis (Table 1). Seven (35.0%) of the data sets were from seven countries in Europe, six (30.0%) data sets were from three countries in the Western Pacific region, two (10.0%) data sets were collected in the United States, and three (15.0%) data sets were from the African region. There were no published data from any country in the Eastern Mediterranean WHO region.

The overall sensitivity and specificity of Xpert HCV viral load testing were 100% (95% CI: 98–100%) and 97% (95% CI: 94–98%), respectively (Figure 4b). Although the sensitivity and specificity were consistently high in analyses stratified by testing year, geographic region, and specimen type, some small but statistically significant differences in estimates were observed. The overall specificity of Xpert HCV viral load testing was 92% (95% CI: 88–95%) in studies conducted between 2012 and 2014 and 100% (95% CI: 99–100%) in studies conducted between 2017 and 2019 (*p* = 0.05) (Table 2). Sensitivity was highest in studies conducted in the Western Pacific region (100%), and specificity was lowest in studies conducted in the South-East Asian region (95%).

Specificity was similar across different specimen types; however, sensitivity showed a small but statistically significant difference for fingerstick compared to serum or plasma (98% vs. 100%, *p* < 0.05) (Figure 4c). Four studies found that fingerstick samples used with a plasma-based cartridge had a pooled sensitivity of 98% (95% CI: 95–99%) and a specificity of 98% (95% CI: 95–99%). The overall sensitivity and specificity were 98% (95% CI: 97–99%) and 99% (95% CI: 98–100%), respectively, for fingerstick. Sensitivity was slightly higher (100% vs. 98%, *p* < 0.001) and specificity slightly lower (97% vs. 100%, *p* < 0.01) for the Xpert HCV viral load test (based on serum/plasma samples) compared to the Xpert HCV VL Fingerprick assay.

**Genedrive HCV ID kit:** Six studies (nine data sets) based on 2470 samples collected in eight countries between 2017 and 2019 were included in the meta-analysis of the Genedrive HCV ID kit, which only utilizes plasma samples (Table 1). The pooled sensitivity and specificity were 99% (95% CI: 98–100%) and 100% (95% CI: 99–100%), respectively (Figure 4d). Five data sets were from five countries in Africa (three countries) and three data sets from Europe (33.3%). There was also one data set collected from India. Specificity was slightly higher in frozen samples than in fresh samples (100% vs. 99%, *p* < 0.001).

**TruenatTM HCV assay:** Two studies and 1181 samples in three countries evaluated the less commonly used TruenatTM HCV assay. The samples were from Spain, India, Ukraine, Georgia, and Thailand. The summary sensitivity was 95% (95% CI: 93–96%) and specificity was 99% (95% CI: 99–100%) between 2017 and 2019 (Figure 4e).

**SAMBA II HCV assay:** Overall, only one study (based on 274 samples collected in Ukraine) evaluated the SAMBA II HCV assay, with a reported sensitivity of 96% (95% CI: 91–98) and a specificity of 100% (95% CI: 97–100%).

### 3.5. Subgroup Analysis by Study Setting, Population, and Specimen Type

Subgroup analyses were conducted on studies stratified according to geographic region, study setting (HCV clinic, drug treatment site, or not reported), population type (PWID, other high-risk, or general population), and specimen types (fingerstick, serum/plasma or whole blood; frozen or fresh). The sensitivity and specificity were similar across different study settings and diverse study populations. However, we found a higher sensitivity (*p* < 0.001) and a lower specificity (*p* < 0.001) in the South-East Asian region than in the European area. Sensitivity was also higher in serum or plasma than in fingerstick capillary whole-blood specimens (for Xpert HCV Viral Load assay: 100% vs. 98%, *p* = 0.02; Truenat and SAMBA: 100% vs. 92%, *p* < 0.001), but there was no difference in specificity. Frozen samples showed a lower specificity than fresh samples (99% vs. 91%, *p* < 0.01) based on the Xpert HCV Viral Load assay; results were similar for the Truenat and SAMBA assays (96% vs. 93%, *p* = 0.03). However, for the Genedrive, the specificity was slightly higher in frozen than fresh samples (100% vs. 99%, *p* < 0.001). However, despite some statistically significant subgroup differences, the overall sensitivity and specificity were high across subgroups.

### 3.6. Grading Quality of Evidence

**Figure 5:**
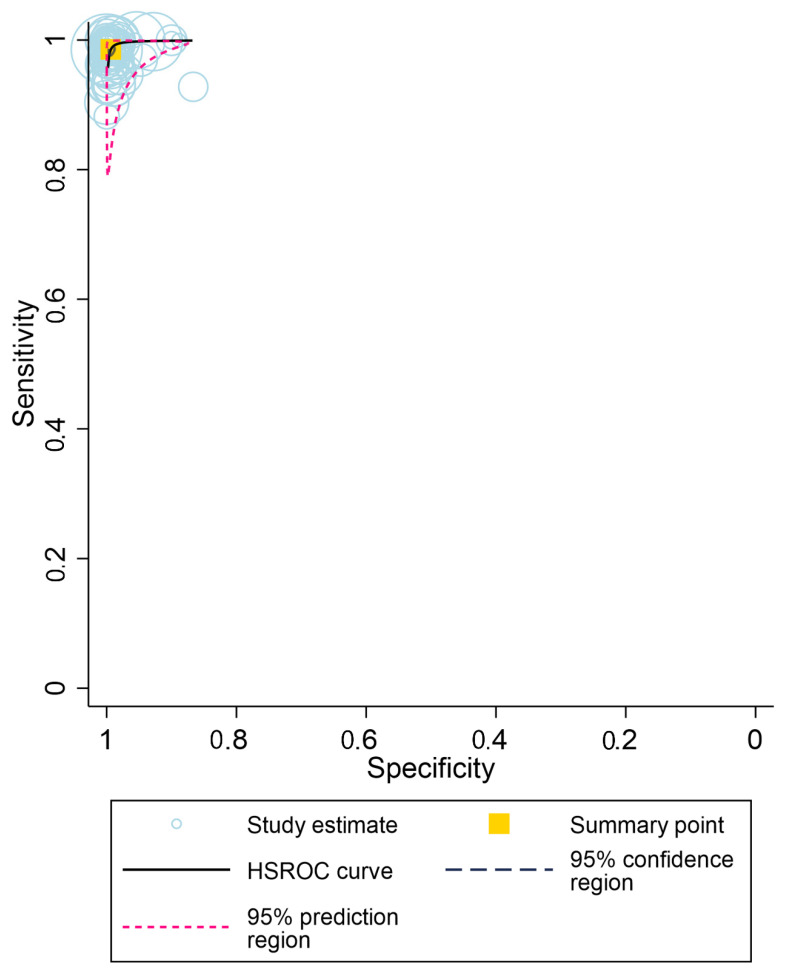
Hierarchical summary receiver-operator curve for the diagnostic accuracy of point-of-care HCV RNA assays used to diagnose chronic HCV infection. (Overall, with all studies combined).

Supplemental Table S5 shows the GRADE assessments for the overall sensitivity and specificity, which were rated overall as moderate-high certainty for both sensitivity and specificity (Figure 5). We downgraded the quality of evidence because of the potential bias in sample collection.

## 4. Discussion

This study evaluated the performance of five different PoC HCV viral load assays compared to conventional laboratory-based testing. In our analysis of 25 studies, the diagnostic performance across PoC HCV viral load assays was very high, with pooled sensitivity and specificities of 99% (95% CI: 98–99%) and 99% (95% CI: 99–100%), respectively. The majority of the studies included in this review were conducted in clinical settings (i.e., HCV clinics and drug treatment sites), with a variety of specimen types (venous whole blood, fingerstick capillary whole blood, and serum or plasma), and in a variety of geographic regions, including both high- and low-income countries. Diagnostic accuracy was consistently high (>90%) across different PoC assays and sample types, clinical settings, and populations, with sensitivity rates ranging from 93% to 100%. However, there were slight differences in estimates in the specific subgroup analyses, especially for sensitivity.

We found that the pooled sensitivity and specificity levels of samples collected through fingerstick sampling were consistently high. This is encouraging, as fingerstick specimen collection is quicker, more straightforward, and more convenient than venipuncture for venous blood samples, especially as venous access may be challenging for persons injecting drugs. Fingerstick sampling can also be used in small primary care clinics, pharmacies, nursing stations, and by unskilled staff [42]. Previous studies indicated that patients prefer fingerstick sampling compared to venipuncture [43]. The high sensitivity and specificity of fingerstick samples may help expand PoC HCV viral load testing and allow testing to be further decentralized, thereby increasing testing and treatment uptake.

Overall, these findings indicate that PoC HCV viral load assays represent a reliable and convenient alternative to laboratory-based assays to confirm HCV viremia and allow prompt linkage to care and treatment in a wide range of settings. While there may be a slight decrease in accuracy in some PoC settings, this disadvantage is likely outweighed by the simplicity of PoC testing and its impact on linkage to and retention in care. The findings of a related systematic review on the effects of using PoC HCV VL on the uptake of HCV RNA testing, HCV treatment, and turnaround time (TAT) provide additional support for the use of PoC viral load assays as a substitute for laboratory-based assays for confirming viremic infection and enabling prompt treatment initiation. The TAT between HCV antibody testing and treatment initiation was 18.5 days [95%CI: 14–53] versus 67 days [95%CI: 50–67] for lab-based high-throughput RNA assays; uptake of treatment increased to 77% (95%CI: 72–83%) versus 53% [95%CI: 31–75%] when using lab-based high-throughput RNA assays [44]. This is particularly relevant in settings with hard-to-reach populations, such as PWID with high HCV prevalence [45,46] and high rates of loss to follow-up, or in rural settings with limited access to laboratory services settings and very limited clinic resources, i.e., in LMICs. The sustained monitoring of PoC assay performance in PoC settings is needed to ensure high accuracy and confirm that the potential benefits related to retention in care occur in clinical practice following widespread implementation.

Our review also highlights some common methodological problems encountered in evaluating diagnostic accuracy. First, most of the included studies were case-control studies, which may introduce a potential risk of bias. Although some statistical heterogeneity was observed using the I^2^ statistic, we did not downgrade for inconsistency, as the I^2^ statistic does not account for threshold effects that occur when evaluating paired measures such as sensitivity and specificity [16], and results were consistent across studies, with all included studies reporting high sensitivity and specificity. Second, most of the participants represented a convenience sample, and the characteristics of the study populations were not always well-reported, which could impact the ability to apply findings to specific populations, clinical situations, and practice settings. Third, most studies were industry-funded, although the companies providing funding were generally not involved in study design or data analysis. Nonetheless, the verification of these findings in non-industry-funded studies would help strengthen the findings. Fourth, most studies included in this review did not disaggregate findings by co-infection status (i.e., HIV and HBV); Refs. [47,48] future studies are needed to examine the diagnostic accuracy of PoC HCV assays in patients with other infectious diseases, particularly HIV-positive individuals. Fifth, we speculated that the detection reagent may exhibit variable sensitivity and specificity for various HCV genotypes, which might be studied further in future investigations. A further limitation is that we focused on the accuracy of PoC assays for the diagnosis of chronic HCV infection and not as a test of cure (e.g., based on a sustained virological response at 12 weeks [SVR12]). A further evaluation of the sensitivity and specificity of PoC HCV viral load testing for SVR12 should be undertaken, particularly for patients who fail treatment with low level viremia. [7]

Our study also has several policy and research implications. It provides evidence to support the inclusion of PoC HCV viral load platforms to enable the diagnosis of HCV viremic infection. The implementation of PoC HCV testing could promote testing and treatment at fully decentralized, co-located HCV testing and treatment centers that include harm reduction services, primary/secondary care, etc. The optimal settings for the provision of HCV PoC platforms are likely to be where there are populations at high risk of loss to follow-up, such as among PWID at harm reduction sites and in prisons; these populations would most benefit from a rapid test-and-treat approach. The availability of multi-disease testing devices and PoC molecular platforms also brings new integration opportunities and may provide significant system efficiencies and cost savings. The WHO recommends PoC RNA assays for other infectious diseases where their use has been established for a longer period of time, such as HIV [49] and TB [50], and they are widely available in LMICs. Finally, although our study demonstrated the diagnostic accuracy of PoC HCV viral load tests, their performance, cost, and accessibility need to be considered when scaling up this service in different settings. Post-marketing monitoring is important to ensure the quality of testing is maintained, that high diagnostic accuracy is achieved in clinical practice, and that the benefits related to increased testing uptake and linkage to care are realized. To further scale up PoC HCV viral load testing, future research should evaluate the impact of PoC HCV viral load testing assays in different clinical settings and identify the main operational considerations and barriers to implementation.

## 5. Conclusions

PoC HCV viral load assays demonstrate excellent diagnostic performance when used in various settings and populations. Based on the evidence in this review, the WHO now recommends using PoC HCV viral load assays as an additional strategy to promote access to confirmatory viral load testing and treatment.

## Figures and Tables

**Table 1:** Summary of characteristics of the 25 included diagnostic performance studies of PoC HCV viral load assays (Gene Xpert, Genedrive, Truenat, and SAMBA).

	Xpert HCV Viral Load (*n* = 16) No. Studies (% of Studies, Total Sample)	Genedrive HCV ID Kit (*n* = 6) No. Studies (% of Studies, Total Sample)	Truenat and SAMBA (*n* = 3) No. Studies (% of Studies, Total Sample)
**Publication year (range)**	2017–2021	2018–2021	2019–2021
**Study setting according to WHO geographic regions**			
European region	7 (35.0%, 1764) ^1^	3 (33.3%, 1707) ^2^	2 (40.0%, 672) ^3^
Western Pacific region	6 (30.0%, 1139)	0 (0.0%, 0)	0 (0.0%, 0)
Americas region	2 (10.0%, 816)	0 (0.0%, 0)	0 (0.0%, 0)
South-East Asian region	3 (15.0%, 451)	1 (11.1%, 320)	2 (40.0%, 696)
African region	2 (10.0%, 385)	5 (55.6%, 869)	0 (0.0%, 0)
Eastern Mediterranean region	0 (0.0%, 0)	0 (0.0%, 0)	0 (0.0%, 0)
Not reported	0 (0.0%, 0)	0 (0.0%, 0)	1 (20.0%, 160)
**Study country income classification ^4^**			
High income	11 (61.1%, 2135) ^5^	1 (11.1%, 1011) ^6^	0 (0.0%, 0)
Upper-middle income	1 (5.6%, 380)	3 (33.3%, 822)	1 (20.0%, 594) ^7^
Lower-middle income	5 (27.8%, 1238)	5 (55.6%, 1063)	3 (60.0%, 774)
Low income	1 (5.6%, 188)	0 (0.0%, 0)	0 (0.0%, 0)
Not reported	0 (0.0%, 0)	0 (0.0%, 0)	1 (20.0%, 160)
**Year of specimen collection**			
2012–2014	2 (12.5%, 783)	0 (0.0%, 0,)	0 (0.0%, 0)
2015–2016	4 (25.0%, 543)	0 (0.0%, 0)	0 (0.0%, 0)
2017–2019	9 (56.3%, 2514)	4 (66.7%, 908)	1 (33.3%, 907)
Not reported	1 (6.3%, 101)	2 (33.3%, 1562)	2 (66.7%, 621)
**Study setting for recruitment**			
HCV clinics	9 (52.9%, 2835) ^8^	2 (28.6%, 449)	1 (33.3%, 907)
Drug treatment sites	6 (35.3%, 803)	2 (28.6%, 696)^9^	0 (0.0%, 0)
Not reported	2 (11.8%, 303)	3 (42.9%, 1325)	2 (66.7%, 621)
**Study design**			
Case-control	1 (6.3%, 101)	3 (50.0%, 1299)	0 (0.0%, 0)
Cross-sectional	7 (43.8%, 2699)	3 (50.0%, 2023)	3 (100.0%, 1528)
Cohort study	8 (50.0%, 1141)	0 (0.0%, 0)	0 (0.0%, 0)
**HCV antibody status and population group**			
Known HCV-positive	6 (37.5%, 1712)	0 (0.0%, 0)	0 (0.0%, 0)
Unknown HCV status	10 (62.5%, 2229)	6 (100.0%, 2470)	3 (100.0%, 1528)
Other high-risk	2 (20.0%, 1156)	1 (16.7%, 425)	1 (25.0%, 313) ^10^
PWID	6 (60.0%, 803)	0 (0.0%, 0)	0 (0.0%, 0)
General population	2 (20.0%, 270)	5 (83.3%, 2045)	3 (75.0%, 1215)
**Specimen type**			
Fingerstick capillary whole blood ^11^	9 (47.4%, 1960) ^12^	0 (0.0%, 0)	2 (28.6%, 1018) ^13^
Serum or plasma	8 (42.1%, 2088)	6 (100.0%, 2470)	3 (42.9%, 1412)
Venous whole blood	2 (10.5%, 1098)	0 (0.0%, 0)	2 (28.6%, 359)
Frozen or fresh samples			
Fresh samples only	11 (68.8%, 2849)	3 (42.9%, 947) ^15^	2 (50.0%, 1067) ^16^
Frozen samples only	3 (18.8%, 424)	4 (57.1%, 1523)	2 (50.0%, 461)
Fresh or frozen samples^14^	1 (6.3%, 614)	0 (0.0%, 0)	0 (0.0%, 0)
Not reported	1 (6.3%, 54)	0 (0.0%, 0)	0 (0.0%, 0)
**Xpert HCV VL Fingerprick assay**			
Yes	6 (33.3%, 1453) ^17^	N/A	N/A
No	12 (66.7%, 2655)	N/A	N/A

^1^ McHugh et al., 2017 [38] provide data from the European and Americas regions; FIND, 2017 [36] provides data from the European, Western Pacific Region, Americas, and African regions; ^2^ Lamoury et al., 2021 [39], FIND, 2018 [40], and Libre et al., 2018 [24] provide data from the European and African regions; ^3^ Two data sets were analyzed by Assennato et al., 2019 [18], one from the European region and the other from Blinded Panel samples; FIND, 2021 [17] provides data from the European and South-East Asian regions; ^4^ From the New World Bank country classifications by income level: 2021–2022 (https://blogs.worldbank.org/opendata/new-world-bank-country-classifications-income-level-2021–2022, accessed on 13 January 2022); ^5^ FIND, 2017 [36] provides data from high-income, upper-middle-income, and lower-middle-income countries; ^6^ Lamoury et al., 2021 [39], and FIND, 2018 [40] provide data from upper-middle-income and lower-middle-income countries; Libre et al., 2018 [24] provide data from high-income and upper-middle-income countries; ^7^ Two series of data were analyzed in Assennato et al., 2019 [18]; one was from lower-middle-income countries and another was analyzed in Blinded Panel samples; FIND, 2021 [17] provides data both from upper-middle-income countries and lower-middle-income countries; ^8^ Studies conducted in Georgia, Cameroon, Greece, and Malaysia by FIND, 2017 [36] that were from HCV clinics, while the study conducted in the United States by FIND, 2017 [36] did not provide any information about the study setting for recruitment; ^9^ Study conducted in Georgia by FIND, 2018 [40] that took place in a harm reduction site, while a study conducted in Cameroon by FIND, 2018 [40] did not provide any information about the study setting for recruitment; ^10^ FIND, 2021 [17] provides data both from other high-risk populations and the general population; ^11^ There are four studies (Grebely et al., 2017 [41], Bregenzer et al., 2019 [32], Calvaruso et al., 2019 [31], and Bielen et al., 2020 [27]) in which fingerprick samples were evaluated with the Xpert HCV Viral Load assay; ^12^ FIND, 2017 [36] provides data both using fingerstick capillary whole-blood and venous whole-blood specimens; Grebely et al., 2017 [41] provide data using both fingerstick capillary whole-blood and plasma specimens; Lamoury et al., 2018 [21] provide data using both fingerstick capillary whole-blood and plasma specimens; ^13^ FIND, 2021 [17] and Assennato et al., 2019 [18] provide data from fingerstick capillary whole-blood, serum or plasma, and venous whole blood specimens; ^14^ No clear documentation of whether fresh or frozen samples were used or the specific number of samples that were fresh or frozen; ^15^ Libre et al., 2018 [24] provide data both using fresh and frozen specimens; ^16^ Assennato et al., 2019 [18] provide data both using fresh and frozen specimens; ^17^ Bregenzer et al., 2019 [32] and Lamoury et al., 2018 [21] provide data both using the Xpert HCV VL Fingerprick assay and the Xpert HCV Viral load assay.

**Table 2:** Diagnostic accuracy of point-of-care HCV RNA assays for diagnosing and monitoring chronic HCV infection in sub-group and meta-regression analyses.

	Xpert HCV Viral Load					Genedrive HCV ID Kit					Truenat and SAMBA				
	Summary Sensitivity % (95% CI)	*p*-Value	Summary Specificity % (95% CI)	*p*-Value	*p* for Joint Model	Summary Sensitivity % (95% CI)	*p*-Value	Summary Specificity % (95% CI)	*p*-Value	*p* for Joint Model	Summary Sensitivity % (95% CI)	*p*-Value	Summary Specificity % (95% CI)	*p*-Value	*p* for Joint Model
Overall	99 (98–99)		99 (98–100)			99 (98–100)		100 (99–100)			95 (94–97)		99 (99–100)		
**Study setting according to WHO geographic regions**															
European region	98 (97–99)	Ref	99 (97–100)	Ref	Ref	99 (97–100)	Ref	100 (99–100)	Ref	Ref	96 (94–98)	Ref	99 (98–100)	Ref	Ref
Western Pacific region	100 (96–100)	<0.001 ***	98 (96–99)	0.42	0.02 *	N/A	N/A	N/A	N/A	N/A	N/A	N/A	N/A	N/A	N/A
Americas region	99 (97–100)	0.04 *	100 (73–100)	0.11	0.16	N/A	N/A	N/A	N/A	N/A	N/A	N/A	N/A	N/A	N/A
South-East Asian region	98 (97–99)	<0.001 ***	99 (97–100)	0.75	0.78	100 (96–100)	0.64	99 (98–100)	0.10	0.94	93 (90–96)	<0.001 ***	99 (98–100)	<0.01 **	0.14
African region	100 (66–100)	0.79	95 (81–99)	<0.01 **	0.28	100 (98–100)	<0.001 ***	100 (98–100)	<0.001 ***	0.13	N/A	N/A	N/A	N/A	N/A
**Study country income classification**															
High income	99 (98–99)	Ref	99 (98–100)	Ref	Ref	99 (97–99)	Ref	100 (99–100)	Ref	Ref	N/A	N/A	N/A	N/A	N/A
Upper-middle income	96 (95–98)	0.08	100 (99–100)	<0.001 ***	0.09	100 (95–100)	0.9	99 (98–100)	<0.001 ***	0.07	96 (94–98)	Ref	99 (99–100)	Ref	Ref
Lower-middle income	99 (97–100)	0.04 *	97 (95–99)	0.7	0.52	100 (96–100)	0.46	100 (99–100)	<0.001 ***	0.2	94 (92–95)	<0.001 ***	99 (97–99)	0.02 *	0.23
Low income	99 (95–100)	0.47	99 (93–100)	0.02 *	0.98	N/A	N/A	N/A	N/A	N/A	N/A	N/A	N/A	N/A	N/A
**Year of specimen collection**															
2012–2014	100 (99–100)	Ref	92 (88–95)	Ref	Ref	N/A	N/A	N/A	N/A	N/A	N/A	N/A	N/A	N/A	N/A
2015–2016	98 (95–100)	<0.001 ***	99 (95–100)	<0.001 ***	<0.01 **	N/A	N/A	N/A	N/A	N/A	N/A	N/A	N/A	N/A	N/A
2017–2019	99 (98–99)	<0.01 **	100 (99–100)	0.03	0.05 *	100 (96–100)	N/A	99 (99–100)	N/A	N/A	95 (94–97)	Ref	99 (99–100)	Ref	Ref
**Study setting for recruitment**															
HCV clinics	99 (98–100)	Ref	99 (97–100)	Ref	Ref	100 (98–100)	Ref	100 (98–100)	Ref	Ref	95 (94–97)	Ref	99 (99–100)	Ref	Ref
Drug treatment sites	99 (97–100)	0.08	99 (98–99)	0.01 **	0.56	100 (95–100)	<0.01 **	99 (98–100)	<0.001 ***	0.19	N/A	N/A	N/A	N/A	N/A
**Study design**															
Cross-sectional	99 (98–99)	Ref	100 (99–100)	Ref	Ref	99 (95–100)	Ref	99 (99–100)	Ref	Ref	95 (94–97)	Ref	99 (99–100)	Ref	Ref
Cohort study	99 (96–99)	0.02 *	98 (96–99)	0.65	0.05 *	N/A	N/A	N/A	N/A	N/A	N/A	N/A	N/A	N/A	N/A
Casecontrol	100 (100–100)	<0.001 ***	100 (100–100)	<0.001 ***	0.3	99 (99–99)	0.84	100 (100–100)	<0.001 ***	0.11	N/A	N/A	N/A	N/A	N/A
**HCV antibody status and Population group**															
Known HCV-positive	100 (96–100)	Ref	94 (90–97)	Ref	Ref	N/A	N/A	N/A	N/A	N/A	N/A	N/A	N/A	N/A	N/A
Unknown HCV status	98 (97–99)	0.95	99 (99–100)	<0.001 ***	0.01 **	99 (98–100)	N/A	100 (99–100)	N/A	N/A	95 (94–97)	Ref	99 (99–100)	Ref	Ref
Other high risk	98 (97–99)	Ref	100 (99–100)	Ref	Ref	97 (92–100)	Ref	100 (99–100)	Ref	Ref	94 (91–96)	Ref	98 (95–100)	Ref	Ref
PWID	99 (97–100)	<0.001 ***	99 (98–99)	0.48	0.25	N/A	N/A	N/A	N/A	N/A	N/A	N/A	N/A	N/A	N/A
General population	100 (100–100)	<0.001 ***	98 (89–100)	<0.001 ***	0.03 *	100 (98–100)	0.18	100 (99–100)	0.31	0.20	96 (94–97)	<0.001 ***	99 (99–100)	<0.01 **	0.26
**Specimen type**															
Fingerstick capillary whole blood	98 (97–99)	Ref	99 (98–100)	Ref	Ref	N/A	N/A	N/A	N/A	N/A	92 (89–95)	Ref	100 (98–100)	Ref	Ref
Serum or plasma	100 (97–100)	0.02 *	96 (93–98)	0.87	0.01 *	99 (98–100)	N/A	100 (99–100)	N/A	N/A	100 (97–100)	<0.001 ***	96 (93–98)	0.55	0.07
Venous whole blood	98 (96–99)	<0.001 ***	100 (98–100)	0.78	0.87	N/A	N/A	N/A	N/A	N/A	96 (93–98)	<0.001 ***	99 (97–100)	0.53	0.34
**Frozen or Fresh samples**															
Fresh samples only	99 (98–99)	Ref	99 (99–100)	Ref	Ref	99 (95–100)	Ref	99 (98–100)	Ref	Ref	96 (94–97)	Ref	99 (99–100)	Ref	Ref
Frozen samples only	100 (55–100)	0.86	91 (81–96)	0.02 *	0.04 *	99 (98–100)	0.48	100 (100–100)	<0.001 ***	0.02 *	93 (88–99)	0.03 *	99 (98–100)	0.16	0.6
Fresh or frozen samples	99 (95–100)	0.98	99 (93–100)	<0.01 **	0.17	N/A	N/A	N/A	N/A	N/A	N/A	N/A	N/A	N/A	N/A
**Xpert HCV VL Fingerprick assay**															
Yes	98 (97–99)	Ref	100 (99–100)	Ref	Ref	N/A	N/A	N/A	N/A	N/A	N/A	N/A	N/A	N/A	N/A
No	100 (98–100)	<0.001 ***	97 (94–98)	0.84	<0.01 **	N/A	N/A	N/A	N/A	N/A	N/A	N/A	N/A	N/A	N/A

* represents < 0.05; ** represents < 0.01; *** represents < 0.001.

## Supplementary Materials

The following supporting information can be downloaded at: https://www.mdpi.com/article/10.3390/diagnostics12051255/s1, Figure S1: QUADAS-2 Tabular Display; Figure S2: Deek’s Funnel plot with superimposed regression line; Table S1: PRISMA-P 2015 CHECKLIST; Table S2: Database search strategy (Terms and synonyms); Table S3: Basic characteristics of evaluated POC viral load assays; Table S4: Summary of study characteristics and diagnostic accuracy of HCV Point-of-care testing assays from 25 studies; Table S5: Quality assessment of diagnostic accuracy studies of HCV viral load testing using the GRADE approach.
