# Supplementary material for: Diagnostic Accuracy of Point-of-Care HCV Viral Load Assays for HCV Diagnosis: A Systematic Review and Meta-Analysis
Source: Diagnostics (Basel). 2022 May 18;12(5):1255. doi: 10.3390/diagnostics12051255 (PMC9141110; doi:10.3390/diagnostics12051255)
Supplement: Supplementary file 1 [file diagnostics-12-01255-s001.zip › diagnostics-1649132-supplementary.pdf]

Supplementary results

Supplementary materials

Supplementary Table S1. PRISMA-P 2015 CHECKLIST.

Supplementary Table S2. Database search strategy (Terms and synonyms).

Supplementary Table S3. Basic characteristics of evaluated POC viral load assays.

Supplementary Table S4. Summary of study characteristics and diagnostic accuracy of HCV Point-of-care testing assays from 25 studies.

Supplementary Figure S1. QUADAS-2 Tabular Display.

Supplementary Figure S2. Deeks' Funnel plot with superimposed regression line.

Supplementary Table S5. Quality assessment of diagnostic accuracy studies of HCV viral load testing using the GRADE approach.

Supplementary Table S1. PRISMA-P 2015 CHECKIST[10]

Section/topic	Item #	Checklist item
**ADMINISTISTRATIVE INFORMATION**		
**Title**		
**Identification**	1a	Identify the report as a protocol of a systematic review.
**Update**	1b	If the protocol is for an update of a previous systematic review, identify as such.
**Registration**	2	If registered, provide the name of the registry (e.g., PROSPERO) and registration number.
**Authors**		
**Contact**	3a	Provide name, institutional affiliation, and email address of all protocol authors; provide physical mailing address of corresponding author.
**Contributions**	3b	Describe contributions of protocol authors and identify the guarantor of the review.
**Amendments**	4	If the protocol represents an amendment of a previously completed published protocol, identify as such a list changes; otherwise, state plan for documenting important protocol amendments.
**Support**		
**Sources**	5a	Indicate sources of financial or other support for the review.
**Sponsor**	5b	Provide name for the review funder and/or sponsor.
**Role of sponsor/Funder**	5c	Describe roles of funder(s), sponsor(s), and/or institution(s), if any, in developing the protocol.
**INTRODUCTION**		
**Rationale**	6	Describe the rationale for the review in the context of what is already known.
**Objectives**	7	Provide an explicit statement of the question(s) the review will address with reference to participants, interventions, comparators, and outcomes (PICO).
**METHODS**		
**Eligibility criteria**	8	Specify the study characteristics (e.g., PICO, study design, setting, time frame) and report characteristics (e.g., years considered, language, publication status) to be used as criteria for eligibility for the review.
**Information sources**	9	Describe all intended information sources (e.g., electronic databases, contact with study authors, trial registers, or other grey literature sources) with planned dates of coverage.
**Search strategy**	10	Present draft of search strategy to be used for at least one electronic database, including planned limits, such that it could be repeated.
**Study records**		
**Data management**	11a	Describe the mechanism(s) that will be used to manage records and data throughout the review.
**Selection process**	11b	State the process that will be used for selecting studies (e.g., two independent reviewers) through each phase of the review (i.e., screening, eligibility, and inclusion in meta-analysis).
**Data collection process**	11c	Describe planned method of extracting data from reports (e.g., piloting forms, done independently, in duplicate), any processes for obtaining and confirming data from investigators.

**Data items**	12	List and define all variables for which data will be sought (e.g., PICO items, funding sources), any pre-planned data assumptions and simplifications.
**Outcomes and prioritization**	13	List and define all outcomes for which data will be sought, including prioritization of main and additional outcomes, with rationale.
**Risk of bias in individual studies**	14	Describe anticipated methods for assessing risk of bias of individual studies, including whether this will be done at the outcome or study level, or both; state how this information will be used in data synthesis.
**Data Synthesis**		
	15a	Describe criteria under which study data will be quantitatively synthesized.
	15b	If data are appropriate for quantitative synthesis, describe planned summary measures, methods of handling data, and methods of combining data from studies, including any planned exploration of consistency (e.g., I2, Kendall's tau).
	15c	Describe any proposed additional analyses (e.g., sensitivity or subgroup analyses, meta-regression).
	15d	If quantitative synthesis is not appropriate, describe the type of summary planned
**Meta-bias(es)**	16	Specify any planned assessment of meta-bias(es) (e.g., publication bias across studies, selective reporting within studies).
**Confidence in cumulative evidence**	17	Describe how the strength of the body of evidence will be assessed (e.g., GRADE).

Supplementary Table S2. Database search strategy (**Terms and synonyms).**

Accuracy	Point-of-Care	HCV	Viral load
Diagnos*	Point-of-care	HCV	Viral load
Accuracy	POC	Hepatitis C	RNA
Performance	Rapid	Virus	Molecular
Evaluat*	Diagnostic*	Chronic	Nucleic acid
Validat*	Near-patient	Liver	NAT
Assess*	Bedside	Infection	Amplification
Sensitivity	On-demand	Disease	PCR
Specificity	Simplified		Polymerase Chain reaction
Agreement	Fingerstick		RT-PCR
Correlation	Fingerprick		Real-time
Clinical	Capillary		Quantitative
Laboratory	Whole blood		Quantification
*Field	Test*		Qualitative
Screening	Assay		Detection
Monitoring	Kit		
	Cepheid		
	GeneXpert		
	Xpert		
	Genedrive		
	Truenat		
	Molbio		
	SAMBA		

Because the above search strategy led to a very narrow search with very few studies in all databases, a simplified search with only the most relevant key words was used.

DATABASE #1: OVID MEDLINE SEARCH DATE: 10/APRIL/2021

	Free-text terms combined with Boolean Operators (AND/OR)	HITS
1	HCV or hepatitis C virus	99.722
2	Point-of-care or POC or rapid diagnostic or near-patient	38.671
3	RNA or viral load or PCR or NAT	1.476.215
4	1 and 2 and 3	126

	RELEVANT STUDIES IDENTIFIED	16

DATABASE #2: EMBASE SEARCH DATE: 11/APRIL/2021

	Free-text terms	HITS
1	HCV or hepatitis C virus	130.479
2	Limit 1 to yr=”2015-Current”	46.060
3	Point-of-care of POC or rapid diagnostic or near-patient	51.203
4	Limit 3 to yr=”2015-Current”	31.005
5	RNA or viral load or PCR or NAT	2.146.318
6	Limit 5 to yr=”2015-Current”	**792.477**
7	2 and 4 and 6	**237**
	**RELEVANT STUDIES IDENTIFIED**	**20**

DATABASE #2: Scholar SEARCH DATE: 14/APRIL/2021

	Free-text terms combined with Boolean Operators (AND/OR)	HITS
1	HCV AND point of care OR rapid test OR near-patient AND RNA OR PCR OR NAT OR viral load;	47.000
6	Limit 5 to yr=”2015-Current”	17.600
7	Limit number of titles screened	1.000
	**RELEVANT STUDIES IDENTIFIED**	**21**

Supplementary Table S3. Basic characteristics of evaluated PoC viral load assays.

Assay short name	Intended use	Sample type	Sample Volume	LoD	Type of result
Xpert HCV VL	Diagnosis and monitoring	Plasma or serum	1,000 µL	10 IU/mL	Quantitative
Xpert HCV VL Fingerstick	Diagnosis and monitoring	Capillary or venous whole blood	100 µL	100 IU/mL	Quantitative
Genedrive HCV	Diagnosis	Plasma	30 µL	2,362 IU/mL	Qualitative
Truenat HCV	Diagnosis and monitoring	Plasma, serum, capillary or venous whole blood	Plasma or serum: 500 µL<br>Whole blood: 250 µL	227.3 IU/mL<br>1,250 IU/mL	Quantitative
SAMBA II HCV	Diagnosis and monitoring	Capillary or venous whole blood	200 µL	653 IU/mL	Qualitative

Supplementary Table S4. Summary of study characteristics and diagnostic accuracy of HCV Point-of-care testing assays from 25 studies.

First Author	Year	Setting	Study Population	Study Period	Study Design	Clinic Setting	HIV co-infection	Specimen Type	Frozen/Fresh Sample	Index Test	Lower Limit of detection	User Type	Turnaround time (min)	Reference Standard	Lower Limit of detection	Sample Size	Indeterminate results	Sensitivity	Specificity	PPV	NPV	Correlation	Bland-Altman	Sources of Funding
McHugh	2017	Europe and the United	HCV-positive patients	2013/8-2014/10	cross-sectional	HCV clinic	N/A	Serum & Plasma	Fresh & Frozen	Xpert® HCV Viral Load assay	10 IU/ml	technicians	105	Abbott RealTime	12 IU/ml	614	32	99.8%	92.8%	97.1%	99.4%	r = 0.989	0.03 log	Commercial
Grebely	2017	Australia	PWID	2016/2-2016/7	Prospective cohort	Drug treatment	N/A	Plasma	Fresh	Xpert HCV viral load	10 IU/ml (LLOQ)	trained member of the clinical	108	Abbott RealTime	12 IU/ml	150	N/A	100.0%	99.1%	97.8%	100.0%	N/A	-0.0357 log	Academic & Commercial
Grebely	2017	Australia	PWID	2016/2-2016/7	Prospective cohort	Drug treatment	N/A	Finger-stick capillary	Fresh	Xpert HCV viral load	10 IU/ml (LLOQ)	trained member of the clinical	108	Abbott RealTime	12 IU/ml	150	N/A	95.5%	98.1%	95.5%	98.1%	N/A	-0.0028 log	Academic & Commercial
Gupta	2017	India	HCV-positive patients	2015/7-2015/12	Prospective observational	HCV clinic	0	Plasma	Frozen	Xpert HCV viral load	4 IU/ml	N/A	105	Abbott RealTime	12 IU/ml	154	N/A	92.81%	86.67%	98.5%	56.5%	R2 = 0.985	0.04 log	Academic
Lamoury	2018	Australia	PWID	2016/8-2016/12	Prospective cohort	Drug treatment	N/A	Plasma	Fresh	Xpert HCV viral load	4 IU/ml	trained member of the clinical	108	Abbott RealTime	N/A	182	2	100.00%	98.29%	97.0%	100.0%	N/A	0.01684 log	Academic & Commercial
Lamoury	2018	Australia	PWID	2016/8-2016/12	Prospective cohort	Drug treatment	N/A	Finger-stick capillary	Fresh	Xpert HCV VL Fingerstick	40 IU/ml	trained member of the clinical	60	Abbott RealTime	N/A	167	18	98.33%	100.00%	100.0%	99.1%	N/A	-0.06761 log	Academic & Commercial
FIND	2018	Georgia	HCV-risk individuals	N/A	Prospective observational	Drug treatment	N/A	Plasma	Fresh	Genedrive HCV ID Kit	N/A	technologists/technicians	45-75	Abbott RealTime	12 IU/ml	270	0	98.36%	99.32%	99.2%	98.7%	N/A	N/A	N/A
FIND	2018	Cameroon	HCV-risk individuals	N/A	Prospective observational	Not reported	N/A	Plasma	Fresh	Genedrive HCV ID Kit	N/A	technologists/technicians	45-75	Abbott RealTime	12 IU/ml	155	0	93.41%	100.00%	100.0%	91.4%	N/A	N/A	N/A
FIND	2017	Georgia	HCV-positive and HCV-risk	2017/8-2018/2	Prospective observational	HCV clinic	0.3%	Finger-stick capillary	Fresh	Xpert HCV VL Fingerstick	N/A	technologists/technicians	<= 60	Abbott RealTime	12 IU/ml	287	N/A	95.80%	100.00%	100.0%	96.0%	N/A	N/A	N/A
FIND	2017	Georgia	HCV-positive and HCV-risk	2017/8-2018/2	Prospective observational	HCV clinic	0.3%	Finger-stick capillary	Fresh	Xpert HCV VL Fingerstick	N/A	technologists/technicians	<= 60	Xpert HCV viral load	4 IU/ml	287	N/A	93.20%	100.00%	100.0%	93.3%	N/A	N/A	N/A
FIND	2017	Georgia	HCV-positive and HCV-risk	2017/8-2018/3	Prospective observational	HCV clinic	0.3%	Venous whole blood	Fresh	Xpert HCV VL Fingerstick	N/A	technologists/technicians	<= 60	Abbott RealTime	12 IU/ml	300	N/A	95.97%	100.00%	100.0%	96.2%	N/A	N/A	N/A
FIND	2017	Georgia	HCV-positive and HCV-risk	2017/8-2018/4	Prospective observational	HCV clinic	0.3%	Venous whole blood	Fresh	Xpert HCV VL Fingerstick	N/A	technologists/technicians	<= 60	Xpert HCV viral load	4 IU/ml	300	N/A	93.46%	100.00%	100.0%	93.6%	N/A	N/A	N/A
FIND	2017	Cameroon	HCV-positive and HCV-risk	2017/10-2018/1	Prospective observational	HCV clinic	6%	Finger-stick capillary	Fresh	Xpert HCV VL Fingerstick	N/A	technologists/technicians	<= 60	Abbott RealTime	12 IU/ml	180	N/A	99.22%	98.08%	99.2%	98.1%	N/A	N/A	N/A
FIND	2017	Cameroon	HCV-positive and HCV-risk	2017/10-2018/2	Prospective observational	HCV clinic	N/A	Finger-stick capillary	Fresh	Xpert HCV VL Fingerstick	N/A	technologists/technicians	<= 60	Xpert HCV viral load	4 IU/ml	182	N/A	99.24%	100.00%	100.0%	98.1%	N/A	N/A	N/A
FIND	2017	Cameroon	HCV-positive and HCV-risk	2017/10-2018/3	Prospective observational	HCV clinic	N/A	Venous whole blood	Fresh	Xpert HCV VL Fingerstick	N/A	technologists/technicians	<= 60	Abbott RealTime	12 IU/ml	194	N/A	97.04%	98.31%	99.2%	93.5%	N/A	N/A	N/A
FIND	2017	Cameroon	HCV-positive and HCV-risk	2017/10-2018/4	Prospective observational	HCV clinic	N/A	Venous whole blood	Fresh	Xpert HCV VL Fingerstick	N/A	technologists/technicians	<= 60	Xpert HCV viral load	4 IU/ml	197	N/A	97.12%	100.00%	100.0%	93.5%	N/A	N/A	N/A
FIND	2017	Greece	HCV-positive and HCV-risk	2017/10-2018/2	Prospective observational	HCV clinic	78%	Finger-stick capillary	Fresh	Xpert HCV VL Fingerstick	N/A	technologists/technicians	<= 60	Abbott RealTime	12 IU/ml	148	N/A	96.34%	100.00%	100.0%	95.7%	N/A	N/A	N/A
FIND	2017	Greece	HCV-positive and HCV-risk	2017/10-2018/3	Prospective observational	HCV clinic	N/A	Finger-stick capillary	Fresh	Xpert HCV VL Fingerstick	N/A	technologists/technicians	<= 60	Xpert HCV viral load	4 IU/ml	148	N/A	97.53%	100.00%	100.0%	97.1%	N/A	N/A	N/A
FIND	2017	Greece	HCV-positive and HCV-risk	2017/10-2018/4	Prospective observational	HCV clinic	N/A	Venous whole blood	Fresh	Xpert HCV VL Fingerstick	N/A	technologists/technicians	<= 60	Abbott RealTime	12 IU/ml	150	N/A	97.59%	98.51%	98.8%	97.1%	N/A	N/A	N/A
FIND	2017	Greece	HCV-positive and HCV-risk	2017/10-2018/5	Prospective observational	HCV clinic	N/A	Venous whole blood	Fresh	Xpert HCV VL Fingerstick	N/A	technologists/technicians	<= 60	Xpert HCV viral load	4 IU/ml	150	N/A	100.00%	100.00%	100.0%	100.0%	N/A	N/A	N/A
FIND	2017	Malaysia	HCV-positive and HCV-risk	2018/1-2018/2	Prospective observational	HCV clinic	4%	Finger-stick capillary	Fresh	Xpert HCV VL Fingerstick	N/A	technologists/technicians	<= 60	Abbott RealTime	12 IU/ml	78	N/A	100.00%	100.00%	100.0%	100.0%	N/A	N/A	N/A
FIND	2017	Malaysia	HCV-positive and HCV-risk	2018/1-2018/2	Prospective observational	HCV clinic	N/A	Finger-stick capillary	Fresh	Xpert HCV VL Fingerstick	N/A	technologists/technicians	<= 60	Xpert HCV viral load	4 IU/ml	77	N/A	100.00%	100.00%	100.0%	100.0%	N/A	N/A	N/A
FIND	2017	Malaysia	HCV-positive and HCV-risk	2018/1-2018/2	Prospective observational	HCV clinic	N/A	Venous whole blood	Fresh	Xpert HCV VL Fingerstick	N/A	technologists/technicians	<= 60	Abbott RealTime	12 IU/ml	80	N/A	100.00%	100.00%	100.0%	100.0%	N/A	N/A	N/A
FIND	2017	Malaysia	HCV-positive and HCV-risk	2018/1-2018/2	Prospective observational	HCV clinic	N/A	Venous whole blood	Fresh	Xpert HCV VL Fingerstick	N/A	technologists/technicians	<= 60	Xpert HCV viral load	4 IU/ml	79	N/A	100.00%	100.00%	100.0%	100.0%	N/A	N/A	N/A
FIND	2017	the United States	HCV-risk individuals	2018/1-2018/2	Prospective observational	N/A	68%	Finger-stick capillary	Fresh	Xpert HCV VL Fingerstick	N/A	technologists/technicians	<= 60	Abbott RealTime	12 IU/ml	193	N/A	100.00%	100.00%	100.0%	100.0%	N/A	N/A	N/A
FIND	2017	the United States	HCV-risk individuals	2018/1-2018/2	Prospective observational	N/A	N/A	Finger-stick capillary	Fresh	Xpert HCV VL Fingerstick	N/A	technologists/technicians	<= 60	Xpert HCV viral load	4 IU/ml	193	N/A	96.67%	100.00%	100.0%	99.4%	N/A	N/A	N/A
FIND	2017	the United States	HCV-risk individuals	2018/1-2018/2	Prospective observational	N/A	N/A	Venous whole blood	Fresh	Xpert HCV VL Fingerstick	N/A	laboratory technician	<= 60	Abbott RealTime	12 IU/ml	202	N/A	100.00%	100.00%	100.0%	100.0%	N/A	N/A	N/A
FIND	2017	the United States	HCV-risk individuals	2018/1-2018/2	Prospective observational	N/A	N/A	Venous whole blood	Fresh	Xpert HCV VL Fingerstick	N/A	laboratory technician	<= 60	Xpert HCV viral load	4 IU/ml	202	N/A	96.77%	100.00%	100.0%	99.4%	N/A	N/A	N/A

First Author	Year	Setting	Study Population	Study Period	Study Design	Clinic Setting	HIV co-infection	Specimen Type	Frozen/Fresh Sample	Index Test	Lower Limit of detection	User Type	Turnaround time (min)	Reference Standard	Lower Limit of detection	Sample Size	Indeterminate results	Sensitivity	Specificity	PPV	NPV	Correlation	Bland-Altman	Sources of Funding
Iwamoto	2019	Cambodia	HCV-positive patients	2017/8-2017/9	Prospective observational	HCV clinic	N/A	Plasma	Fresh	Xpert HCV viral load	4.91 IU/ml	technicians	< 120	HCV Viral Load Cobas	N/A	590	N/A	100.0%	95.4%	98.7%	100.0%	r = 0.94	-0.01 log	N/A
WHO PQ	2017	Australia	General population	N/A	Retrospective cohort	N/A	N/A	Serum and plasma	Frozen	Xpert HCV viral load	4.91 IU/ml	N/A	105	HCV Viral Load Cobas	N/A	101	1	100.0%	100.0%	100.0%	100.0%	N/A	-0.135 log	N/A
Wlassow	2019	France	General population	2012/9-2013/11	Retrospective observational	HCV clinic	N/A	Venous whole blood	Frozen	Xpert HCV viral load	N/A	N/A	105	Xpert HCV viral load	N/A	169	1	100.0%	90.0%	96.0%	100.0%	r = 0.80	1.93 log	Academic
Bregenzer	2019	Swtizerland	HCV-risk individuals	2016/11-2018/10	Prospective observational	HCV clinic	39%	Finger-stick capillary	Fresh	Xpert HCV viral load	4.0 IU/ml (plasma)	nurses	105	HCV Viral Load Cobas	12 IU/ml	194	N/A	97.0%	94.7%	95.0%	96.8%	R2 = 0.9165	1.322 log	Commercial
Bregenzer	2019	Swtizerland	HCV-risk individuals	2018/11-2019/5	Prospective observational	HCV clinic	13%	Finger-stick capillary	Fresh	Xpert HCV VL Fingerstick	40 IU/ml	nurses	60	HCV Viral Load Cobas	13 IU/ml	33	N/A	100.0%	88.9%	88.2%	100.0%	R2 = 0.9899	0.104 log	Commercial
Calvaruso	2019	Italy	HCV-positive patients	2016/9-2016/12	Prospective observational	HCV clinic	N/A	Finger-stick capillary	Fresh	Xpert HCV viral load	N/A	nurses	120	TaqMan Real time	15 IU/ml	57	2	100.0%	100.0%	100.0%	100.0%	r = 0.809	N/A	Academic
Saludes	2020	Spain	PWID	2018/5-2019/2	Prospective observational	Drug treatment	N/A	Finger-stick capillary	Fresh	Xpert HCV VL Fingerstick	35 IU/ml	Trained nursing DCR staff	58	Xpert HCV viral load	12 IU/ml	100	0	98.4%	100.0%	100.0%	97.4%	r2 = 0.8806	-0.0659 log	Academic
Grebely	2020	Australia	PWID	2017/5-2018/3	Non-randomized intervention	Drug treatment	0	Finger-stick capillary	Fresh	Xpert HCV VL Fingerstick	N/A	N/A	58	Aptima HCV Quant Dx	12 IU/ml	36	17	100.0%	95.7%	92.9%	100.0%	N/A	N/A	Academic
Mohamed	2020	Tanzania	PWID	2018/12-2019/2	Prospective observational	Drug treatment	36%	Finger-stick capillary	Fresh	Xpert HCV VL Fingerstick	40 IU/ml	laboratory technician	60	Xpert HCV viral load	12 IU/ml	188	12	99.1%	98.7%	99.1%	98.7%	r2 = 0.95	0.13 log	Academic
Bielen	2020	Belgium	PWID	2018/1-2019/3	Non-randomized intervention	Drug treatment	0.70%	Finger-stick capillary	Fresh	Xpert HCV viral load	10 IU/ml	trained member of the clinical	108	Artus HCV RNA kit	12 IU/ml	147	6	100.0%	99.1%	97.2%	100.0%	N/A	-0.76 log	Academic
Thedja	2021	Indonesia	HCV-positive patients	2018/6-2019/1	Prospective observational	HCV clinic	N/A	Plasma	Fresh	Xpert HCV viral load	4 IU/ml	N/A	105	HCV Viral Load Cobas	12 IU/ml	243	0	100.0%	98.4%	99.4%	100.0%	r = 0.97	-0.25 log	Academic
Jasirwan	2020	Indonesia	HCV-positive patients	2017/03-2017/11	Prospective observational	HCV clinic	N/A	Plasma	N/A	Xpert HCV viral load	N/A	N/A	105	HCV Viral Load Cobas	12 IU/ml	54	0	100.0%	90.0%	97.8%	100.0%	r = 0.993	<1 log	Academic
Libre	2018	European countries	General population	N/A	Retrospective cohort	N/A	3.10%	Serum and plasma	Frozen	Genedrive HCV ID Kit	N/A	N/A	88	Abbott RealTime	12 IU/ml	915	10	98.6%	100.0%	100.0%	98.8%	r = 0.72 (UK)	-0.06 log (UK)	Academic
Libre	2018	European countries	General population	N/A	Retrospective cohort	N/A	3.10%	Serum and plasma	Fresh	Genedrive HCV ID Kit	N/A	N/A	88	Abbott RealTime	12 IU/ml	96	4	98.0%	100.0%	100.0%	97.9%	r = 0.73 (UK)	-0.07 log (UK)	Academic
Libre	2018	South Africa	General population	N/A	Retrospective cohort	N/A	N/A	Serum and plasma	Frozen	Genedrive HCV ID Kit	N/A	N/A	88	Abbott RealTime	12 IU/ml	126	4	100.0%	100.0%	100.0%	100.0%	N/A	N/A	Academic
WHO PQ	2020	Nigeria	General population	2019/4-2019/12	Retrospective cohort	HCV clinic	N/A	Plasma	Frozen	Genedrive HCV ID Kit	N/A	N/A	90	HCV Viral Load Cobas	N/A	129	2	98.8%	100.0%	100.0%	98.0%	N/A	N/A	N/A
Padhi	2020	India	General population	2017/10-2019/2	Prospective observational	HCV clinic	0	Plasma	Frozen	Genedrive HCV ID Kit	3.37 log10 IU/ml (2362	N/A	90	Abbott RealTime	12 IU/ml	320	0	100.0%	100.0%	100.0%	100.0%	N/A	N/A	Academic
Lamoury	2021	Georgia, Cameroon	General population	2019/6-2019/10	Prospective observational	Drug treatment	1.20%	Plasma	Fresh	Genedrive HCV ID kit	N/A	nurses & laboratory	90	Abbott RealTime	12 IU/ml	426	0	96.2%	99.5%	99.5%	96.4%	N/A	N/A	Academic
Lamoury	2021	Georgia, Cameroon	General population	2019/6-2019/11	Prospective observational	Drug treatment	1.20%	Plasma	Fresh	Genedrive HCV ID kit	N/A	nurses & laboratory	90	Abbott RealTime	12 IU/ml	426	0	100.0%	99.5%	99.5%	100.0%	N/A	N/A	Academic
Lamoury	2021	Georgia, Cameroon	General population	2019/6-2019/12	Prospective observational	Drug treatment	1.20%	Plasma	Fresh	Genedrive HCV ID kit	N/A	nurses & laboratory	90	Abbott RealTime	12 IU/ml	426	0	100.0%	98.7%	98.5%	100.0%	N/A	N/A	Academic
Mboup	2019	Senegal	General population	2018/11-2019/1	Retrospective cohort	N/A	N/A	Plasma	Frozen	Genedrive HCV ID kit	N/A	N/A	< 90	Abbott RealTime	12 IU/ml	33	N/A	100.0%	100.0%	100.0%	100.0%	N/A	N/A	Commercial
Mehta	2020	India	General population	N/A	Retrospective observational	N/A	N/A	Plasma	Frozen	TruenatTM HCV assay	N/A	N/A	N/A	Abbott RealTime	12 IU/ml	347	N/A	95.0%	98.8%	98.8%	94.9%	R2 = 0.893	0.88 IU/ml	Academic
Assennato	2019	Blinded Panel samples	General population	N/A	Retrospective observational	N/A	N/A	Plasma	Fresh	SAMBA II HCV	N/A	N/A	N/A	HCV Viral Load Cobas	12 IU/ml	160	0	97.5%	100.0%	100.0%	93.0%	N/A	N/A	Commercial
Assennato	2019	Ukraine	General population	N/A	Retrospective observational	N/A	N/A	Venous or finger-prick	Fresh	SAMBA II HCV	N/A	N/A	N/A	HCV Viral Load Cobas	12 IU/ml	114	0	88.2%	100.0%	100.0%	95.2%	N/A	N/A	Commercial
FIND	2021	Georgia	General population	2020/1-2020/7	N/A	N/A	N/A	Finger-stick capillary	Fresh	TruenatTM HCV assay	N/A	N/A	N/A	Abbott RealTime	N/A	242	N/A	96.7%	99.3%	98.9%	98.0%	N/A	N/A	N/A
FIND	2021	Georgia	General population	2020/1-2020/7	N/A	N/A	N/A	Plasma	Fresh	TruenatTM HCV assay	N/A	N/A	N/A	Abbott RealTime	N/A	245	N/A	96.8%	99.3%	98.9%	98.1%	N/A	N/A	N/A
FIND	2021	Georgia	General population	2020/1-2020/7	N/A	N/A	N/A	Venous whole blood	Fresh	TruenatTM HCV assay	N/A	N/A	N/A	Abbott RealTime	N/A	245	N/A	96.8%	98.7%	97.8%	98.0%	N/A	N/A	N/A
FIND	2021	Georgia	General population	2020/1-2020/7	N/A	N/A	N/A	Serum	Fresh	TruenatTM HCV assay	N/A	N/A	N/A	Abbott RealTime	N/A	244	N/A	97.8%	100.0%	100.0%	98.7%	N/A	N/A	N/A
FIND	2021	Georgia	General population	2020/1-2020/7	N/A	N/A	N/A	Venous whole blood	Fresh	TruenatTM HCV assay	N/A	N/A	N/A	TruenatTM HCV assay	N/A	241	N/A	98.9%	98.7%	97.8%	99.3%	N/A	N/A	N/A
FIND	2021	Georgia	General population	2020/1-2020/7	N/A	N/A	N/A	Serum	Fresh	TruenatTM HCV assay	N/A	N/A	N/A	TruenatTM HCV assay	N/A	244	N/A	98.9%	99.4%	98.9%	99.4%	N/A	N/A	N/A
FIND	2021	Ukraine	HCV-risk individuals	2020/9-2021/3	N/A	N/A	N/A	Finger-stick capillary	Fresh	TruenatTM HCV assay	N/A	N/A	N/A	N/A	N/A	313	N/A	93.0%	98.8%	99.5%	83.8%	N/A	N/A	N/A
FIND	2021	Ukraine	HCV-risk individuals	2020/9-2021/3	N/A	N/A	N/A	Plasma	Fresh	TruenatTM HCV assay	N/A	N/A	N/A	N/A	N/A	312	N/A	94.7%	97.6%	99.1%	87.2%	N/A	N/A	N/A
FIND	2021	Thailand	HCV-positive and HCV-risk	2020/7-2020/12	N/A	N/A	N/A	Finger-stick capillary	Fresh	TruenatTM HCV assay	N/A	N/A	N/A	N/A	N/A	349	N/A	90.4%	100.0%	100.0%	80.5%	N/A	N/A	N/A
FIND	2021	Thailand	HCV-positive and HCV-risk	2020/7-2020/12	N/A	N/A	N/A	Plasma	Fresh	TruenatTM HCV assay	N/A	N/A	N/A	N/A	N/A	348	N/A	93.2%	99.0%	99.6%	85.1%	N/A	N/A	N/A

Supplementary Figure S1. QUADAS-2 Tabular Display.

	Risk of Bias				Applicability Concerns		
	Patient Selection	Index Test	Reference Standard	Flow and Timing	Patient Selection	Index Test	Reference Standard
Assennato 2019	−	?	?	?	?	?	+
Bielen 2020	−	+	?	+	?	+	?
Bregenzer 2019	−	+	+	+	+	+	+
Calvaruso 2019	−	+	+	?	?	+	+
FIND 2017	+	+	+	+	+	+	+
FIND 2018	+	+	+	+	+	+	+
FIND 2021	+	+	+	+	+	+	+
Grebely 2017	+	+	+	?	+	+	+
Grebely 2020	−	?	?	?	+	+	?
Gupta 2017	−	?	?	?	+	+	+
Iwamoto 2019	−	+	+	+	+	+	+
Jasirwan 2020	−	?	?	?	+	+	+
Lamoury 2018	+	+	+	?	+	+	+
Lamoury 2021	+	+	+	+	+	+	+
Libre 2018	?	+	+	?	?	+	+
Mbout 2021	?	?	−	?	+	?	?
McHugh 2017	−	+	?	+	+	+	+
Mehta 2020	+	?	?	+	?	?	?
Mohamed 2020	−	+	?	+	+	+	+
Padhi 2020	?	?	+	?	+	+	+
Saludes 2020	−	?	?	+	+	+	+
Thedja 2021	−	?	?	?	+	+	+
WHO 2017	?	?	?	?	?	+	+
WHO 2020	?	?	?	?	?	+	+
Wlassow 2019	?	?	?	?	+	+	+

− High ? Unclear + Low

Supplementary Figure S2. Deeks' Funnel plot with superimposed regression line.

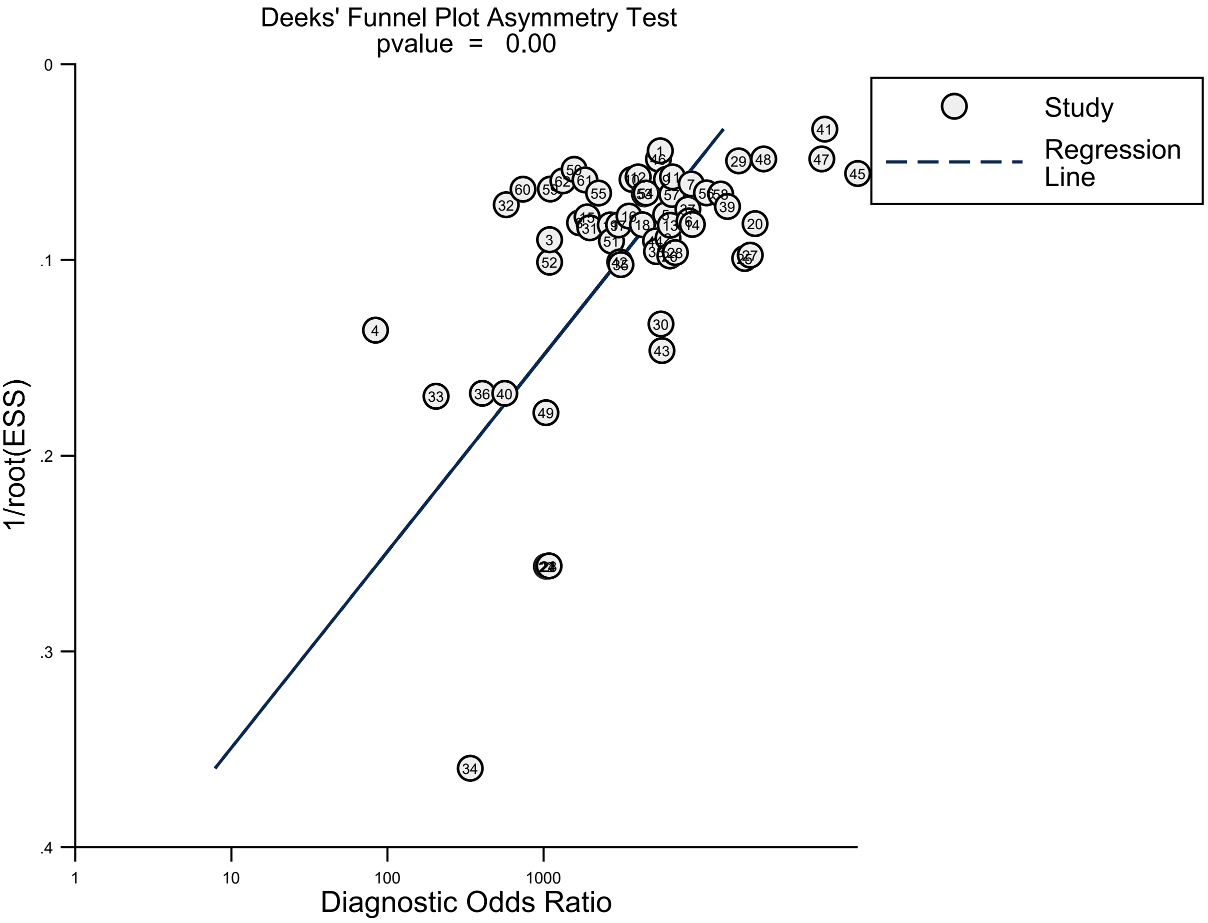

Supplement Table S5. Quality assessment of diagnostic accuracy studies of HCV viral load testing using the GRADE approach.

No. of studies	Design	GRADE components					
		**Risk of bias**	**Indirectness**	**Inconsistency**	**Imprecision**	**Other Considerations**	**Overall Certainty**
**Sensitivity of the HCV viral load test**							
25 studies (7592 patients)	Observational studies	Not serious[1]	Not serious	Not serious	Not serious	Some additional concerns[2]	Moderate-High
**Specificity of the HCV viral load test**							
25 studies (7592 patients)	Observational studies	Not serious[1]	Not serious	Not serious	Not serious	Some additional concerns[2]	Moderate-High

[1] We examined the study conclusions stratified by the risk of bias (high risk of bias versus low or unclear) and found that the overall findings were robust when stratified according to use of appropriate methods to select patients.

[2] Several studies were funded by diagnostic companies, and this may have influenced our results (especially small sample effect and publication bias).
